# Molecular biology research in neuropsychiatry: India’s contribution

**DOI:** 10.4103/0019-5545.69223

**Published:** 2010-01

**Authors:** T. S. Sathyanarayana Rao, B. N. Ramesh, P. Vasudevaraju, K. S. J. Rao

**Affiliations:** Department of Psychiatry, JSS Medical College, JSS University, Mysore, India; 1 Biochemistry and Nutrition, CFTRI, CSIR Unit, Mysore, India

**Keywords:** Depression, bipolar disorders, sexual dysfunction, autism, dementia, trace metals, DNA conformation, DNA stability, cell death, D1 receptors, genes, pedigree, enzymes, diet, mutations

## Abstract

Neuropsychiatric disorders represent the second largest cause of morbidity worldwide. These disorders have complex etiology and patho-physiology. The major lacunae in the biology of the psychiatric disorders include genomics, biomarkers and drug discovery, for the early detection of the disease, and have great application in the clinical management of disease. Indian psychiatrists and scientists played a significant role in filling the gaps. The present annotation provides in depth information related to research contributions on the molecular biology research in neuropsychiatric disorders in India. There is a great need for further research in this direction as to understand the genetic association of the neuropsychiatric disorders; molecular biology has a tremendous role to play. The alterations in gene expression are implicated in the pathogenesis of several neuropsychiatric disorders, including drug addiction and depression. The development of transgenic neuropsychiatric animal models is of great thrust areas. No studies from India in this direction. Biomarkers in neuropsychiatric disorders are of great help to the clinicians for the early diagnosis of the disorders. The studies related to gene-environment interactions, DNA instability, oxidative stress are less studied in neuropsychiatric disorders and making efforts in this direction will lead to pioneers in these areas of research in India. In conclusion, we provided an insight for future research direction in molecular understanding of neuropsychiatry disorders.

## INTRODUCTION

Neuropsychiatric disorders represent the second largest cause of morbidity worldwide. These disorders have complex etiology. The genetic linkage is only 10% while remaining 90% are sporadic in nature. The World Health Organization has estimated that neuropsychiatric disease burden comprises 13% of all reported diseases. The psychiatric disorders include major depression, anxiety, schizophrenia, bipolar disorder, obsessive-compulsive disorder, alcohol and substance abuse, and attention-deficit hyperactivity disorder. Approximately one in five Americans experience an episode of a psychiatric illness such as schizophrenia, mood disorder (depression and bipolar disorder) or anxiety and a similar situation is predicted to be prevailing in developing countries too. The Diagnostic and Statistical Manual of Mental Disorders (DSM-IV) defines the criteria for a wide array of mental illnesses. Most Psychiatrists use it as a basis for diagnosis. But diagnosis is still a challenge as many disorder episodes are overlapping.

The prevalence of disorders, and their economics and societal aspects has to be understood through research programs aimed at elucidating the etiologies and pathophysiological mechanisms of these devastating disorders. The final goal will be clear diagnosis, management and drug discovery.

### Major depression

Major depression is an affective disorder and the symptoms include feelings of profound sadness, worthlessness, despair and loss of interest in all pleasures. The individuals having depression also experience mental slowing, a loss of energy and an inability to make decisions or concentrate. The symptoms can range from mild to severe, and are often associated with anxiety and agitation.[1] Worldwide, its prevalence is 21% in women and 13% in men. Its occurrence is two to three times more common in first-degree relatives of depressed persons, suggesting a genetic predisposition.

### Bipolar disorder

Bipolar disorder is a chronic mental illness, which begins, in adolescence or early adulthood. The typical bipolar disorders initiates between 15 and 25 years. The bipolar disorder onsets with initial episode of depression; these episodes of depression go as undiagnosed and are not treated appropriately. Bipolar Disorder includes Major Depressive Episodes and Hypomanic episodes. The significant portion of people suffering from manic depression did not have full manic episodes. The classification was divided into Bipolar I and Bipolar II. However, Bipolar II is often a first step to Bipolar I. It is seen that over five years, between 5 and 15% of those with Bipolar II change diagnosis to Bipolar I. Approximately 0.5% of people develop Bipolar II in their lifetimes. The psychobiology of therapeutic approach to anxiety disorders is still complex.[2]

### Cyclothymic disorder

Cyclothymic disorder is characterized by alternating hypomania and depressive episodes. Like bipolar, cyclothymia involves cycling between highs and lows, but it never reaches full mania or major depression. It was earlier referred to as a cycloid personality. Over a lifetime, the chances of having Cyclothymic disorder are from 0.4 to 1%.

### Schizophrenia

Schizophrenia is a neuropscychiatric disorder with multiple predisposing genes having variable contributions. Avasthi and Singh[3] reviewed Schizophrenia relation: Indian scenario in last decade. The clinical management of Schiziphrenia through multiple drugs has been understood critically by various groups.[4] Bhargava and Sethi[5] highlighted the role of transexualism and schizophrenia and its differential response to drugs. Many of the drugs currently marketed for the treatment of schizophrenia are dopamine D2 receptor antagonists or partial agonists with or without mixed receptor pharmacology. These primarily treat the positive symptoms of schizophrenia. Nikam and Avasthi[6] reviewed therapeutics of Schizophrenia. They cited increase in atypical antipsychotics leading to increased tolerability compared with typical antipsychotics. Atypical antipsychotics show extra pyramidal side effects and D2 receptor antagonism, and an increased efficacy of the treatment of the negative symptoms of schizophrenia. There is strong evidence that genetic factors play a key role in contributing to the etiology of autism, schizophrenia and bipolar disorders, with genetics link being 80% for each. These disorders are known to have complex inheritance pattern with multiple genetic and environmental factors, but the pattern of inheritance regarding Schizophrenia is complex.[7] The neuropsychiatric disorders have complex genetics, which further make it complex in the form of phenotypic complexities. Autism, schizophrenia and bipolar disorder are a group of neuropsychiatric disorders with symptoms that define groups of patients with broadly similar outcomes and responses to treatment. The diagnostic categories, mostly heterogeneous, and the boundaries between them are seen to be arbitrary. The whole-genome technologies have discovered rare copy number variants and common single-nucleotide polymorphisms. The single-nucleotide polymorphisms are associated with risk of developing neuropsychiatric disorders.[8] There is an overlap between the genetic loci and even alleles that predispose to the different phenotypes. The copy number variations are found to be important risk factors for autism and schizophrenia. The conclusion from the study is that specific genetic loci implicated to encode proteins neurexins and neuroligins. These proteins play a crucial role in synaptic development and plasticity.

### Dementia

Dementia is mental disorder, which is characterized by loss of memory and cognition. An international team including India made significant contribution on the dementia rate in developed and developing countries. The one-phase cross-sectional surveys of all residents aged 65 years and older (n = 14 960) in 11 sites in seven low-income and middle-income countries (China, India, Cuba, Dominican Republic, Venezuela, Mexico, and Peru) was investigated. The prevalence of dementia varied widely, from 0.3% (95% CI 0.1-0.5) in rural India to 6.3% (5.0-7.7) in Cuba. After standardization for age and sex, DSM-IV, the prevalence in urban Latin American sites was four-fifths of that in Europe (standardized morbidity ratio 80 [95% CI 70-91]). In China the prevalence was only half in India and, rural Latin America, a quarter or less of the European prevalence in rural India). The 10/66-dementia prevalence was higher than DSM-IV dementia, and more consistent across all sites varying between 5.6% (95% CI 4.2-7.0) in rural China and 11.7% (10.3- 13.1) in the Dominican Republic. The validity of the 847 of 1345 cases of 10/66 dementia not confirmed by DSM-IV was supported by high levels of associated disability (mean WHO Disability Assessment Schedule II score 33.7 [SD 28.6]). This indicates that the DSM-IV dementia criterion might underestimate dementia prevalence, especially in regions with low awareness of this emerging public-health problem.[9]

There are a few reports on the clinical profiles of young-onset dementia from India. Nandi *et al*.[10] conducted a study to determine the clinical profile of patients attending a specialist cognitive disorders clinic in West Bengal. Almost one-fourth (94/379, 24.5%) of all the patients with dementia were of young onset. Women constitute about one-third of these cases. There was a gradual increase in the number of cases with rising age. The most common etiologies were Alzheimer disease (33%), frontotemporal dementia (27%), and vascular dementia (20%). A positive family history was found in close to one-fifth of the patients.

There is an international study linking a relationship between diet and dementia. Albanese *et al*.[11] found that dietary intake and the prevalence of dementia varied between studied sites. The combined site-specific Poisson regression prevalence ratios (PRs) for the association between fish and meat consumption and dementia in 2 fixed-effect model meta-analyzee was adjusted for socio-demographic and health characteristics and fish and meat consumption. They found a dose-dependent inverse association between fish consumption and dementia (PR: 0.81; 95% CI: 0.72, 0.91). The result was consistent across all sites except India and a less-consistent, dose-dependent, direct association between meat consumption and prevalence of dementia (PR: 1.19; 95% CI: 1.07, 1.31). Further, Das *et al*.[12] has contributed significantly in area of dementia research in India.

The amnestic and multiple domain types dementia are two types of dementia among nondemented and nondepressed elderly subjects aged 50 and older. This is a cross sectional community screening study. The subjects are selected by systematic random sampling for the assessment of cognitive function with the help of a validated cognitive questionnaire battery. The results indicated that prevalence of MCI detected based on neuropsychological testing is 14.89% (95% CI: 12.19 to 17.95). The prevalence of the amnestic type is 6.04% (95% CI: 4.40 to 8.1). The multiple domain type is 8.85% (95% CI: 6.81 to 11.32). The data insights that the amnestic type is more common among men and the multiple domain type among women with advancement of age. This is the first study on the prevalence of amnestic type in developing countries. Also, Das *et al*.[13] has undertaken a cross-sectional population-based epidemiological study. The study was aimed to understand the prevalence of epilepsy, stroke, dementia and Parkinsonism in the city of Kolkata, India. 52,377 subjects involved in the study. This study indicates high rate of stroke and overall lower prevalence of Parkinsonism and dementia compared to western countries. Recently, Das *et al*.[14] also reported the prevalence of common neurological disorders among the elderly population in the city of Kolkata using cross-sectional study. The results indicated that the prevalence rates (per 1000 elderly population - ≥ 60years) of following disorders in decreasing order: Stroke - 33.39, essential tremor - 13.76, dementia - 7.89, Parkinsonism - 3.30 and epilepsy - 2.57. The study insights show the prevalence of high risk for stroke among elderly population in India.

Rao *et al*.[15] reviewed critically the role of nutrition in the depression and other mental illness. Dietary deficiencies of antioxidants and nutrients during aging precipitate the brain diseases, which may be due to failure of protective mechanisms. Diet containing proteins rich in essential amino acids have a beneficial effect in keeping away the onset of depression. A diet rich in docosahexanoic acid and eicosapentaenoic acid found in fish oil have anti-depressant effect on humans. These findings may have greater impact on the therapeutic intervention of neuropyschiatric disorders. Very recently, Ramesh *et al*.[16] reported a link between dietary factors and the risk of developing Alzheimer’s disease (AD). They also discussed the role of environmental factors, dietary protective and risk factors for AD. Diets rich in saturated fatty acids and alcohol; and deficient in antioxidants and vitamins appear to promote the onset of the disease, while diets rich in unsaturated fatty acids, vitamins, antioxidants, and wine likely suppress its onset. Evidence suggests that diets rich in polyphenols and some spices suppress the onset of AD by scavenging free radicals and preventing oxidative damage. Specific metal chelators have been tested for therapy, but not been very successful, probably, due to late administration after triggering of brain damage. Since several dietary polyphenols are known to chelate metals, their routine use may also be protective against the onset of AD.

### Autism

Autism spectrum disorder (ASD) is a of complex neurodevelopment disorders, characterized by social impairments, communication difficulties. It is also characterized by abnormal behavior such as restricted, repetitive, and stereotyped behavioral patterns. The genetic imprinting of certain genes is reported in the autism. The paternal imprinting of HTR2A with expression from only one allele is reported. There are no reports on HTR2A and its association with neuropsychiatric disorders from Indian population. The study showed an association of the above-mentioned markers of HTR2A with Autism spectrum disorder (ASD) in Indian population. The genotyping analyses are carried out for probands, parents and controls. The results indicated that HTR2A is unlikely to be a genetic marker for ASD in Indian population.[17] Recently, Guhathakurta *et al*.[18] showed an association of a VNTR of 17 bp at intron2 (STin2) and an SNP at 3’UTR (HTT-3’UTR-SNP) of the gene with autism using family and population-based approaches. It is concluded that specific haplotypes of the two markers (LRS = 11.85, p(c) = 0.02), in autistic cases suggests that either these markers or nearby markers of SLC6A4 that are in LD. Still there is a great need to understand genomics and proteomics of Dementia and Autism.

### Sexual dysfunction: Biology of sexual dysfunction less studied all over the world

Sexual dysfunction is a major risk factor for anxiety and depression. The reports of the prevalence of female sexual dysfunction (FSD) are scant. Recently, Singh *et al*.[19] conducted a cross-sectional survey in a medical outpatient clinic of a tertiary care hospital, Vellore, India. The aim of the study was to map the prevalence and risk factors for FSD. The results indicated that FSFI total scores suggested FSD in two-thirds of the 149 women (73.2%; 95% confidence intervals [CI] 65.5% to 79.6%). FSFI domain scores suggested difficulties with desire in 77.2%; arousal in 91.3%; lubrication in 96.6%; orgasm in 86.6%, satisfaction in 81.2%, and pain in 64.4%. Age above 40 years (odds ratios [OR] 11.7; 95% CI 3.4 to 40.1) and fewer years of education (OR 1.2; 95% CI 1.0 to 1.3) were identified by logistic regression as contributory.

Also, Kumar *et al*.[20] examined the association between alpha-blocker use and sexual dysfunction among men. The use of alpha-adrenergic receptor blocking agents results in an improvement in LUTS for many men. The use of alpha-blockers for LUTS was associated with a decreased risk of sexual dysfunction. Recently, Newman *et al*.[21] conducted a study on 200 men who have sex with men (MSM) in public sex environments (PSEs) in Chennai, India. One-third of the studied had unprotected receptive anal sex (URAS) the last time and 36% reported inconsistent condom use in the past month. URAS was found associated with younger age, less than high school education, low income, and low HIV transmission knowledge (adjusted odds ratio [AOR] = 2.1, 2.5, 3.7 and 2.5, respectively). Erectile dysfunction is defined as the persistent inability to achieve or maintain an erection adequate for satisfactory sexual activity. The prevalence increases with age.

Erectile dysfunction (ED) is a complex condition where in men with minimal organic ED may develop a variable degree of psychogenic component sufficient to reduce the efficacy of medical management. Taneja[22] studied combination medicine in ED. It is concluded that the priming the patients with trazodone appears to be a reasonably good alternative in patients who have initial failure to oral sildenafil citrate and have been found to have no organic cause of ED. There are problems in management of sexual dysfunction by drugs.[23] There is a great need to understand the biochemistry and molecular biology of sexual dysfunction.

### Role of molecular biology in neuropsychiatric disorders in India

To understand the genetic association of the neuropsychiatric disorders, molecular biology has a tremendous role to play. For example, to understand the inheritance pattern of the particular gene associated with the particular disorder, research in of molecular biology is essential. For example, the role of chromatin modulation in depression is new concept. The alterations in gene expression are implicated in the pathogenesis of several neuropsychiatric disorders, including drug addiction and depression. The development of transgenic neuropsychiatric animal models is a great thrust area. No studies from India in this direction. Biomarkers in neuropsychiatric disorders are of great help to the clinicians for the early diagnosis of the disorders. Gene-environment interactions, DNA instability, oxidative stress are less studied in neuropsychiatric disorders and making efforts in this direction will lead to pioneers in these areas of research in India. The molecular biology of the above mentioned areas would help to understand the neuropsychiatric disorders.

In recent years, it has become widely recognized that a comprehensive understanding of chromatin biology is necessary to better appreciate its role in a wide range of diseases.[24] The epigenetic regulation of gene expression refers to the chromatin modifications that occur at the level of DNA, and protein. The epigenetic regulatory machinery which includes DNA methylation and histone acetylation/deacetylation studied in depth. The results indicate that the inhibitors of DNA methyltransferases (DNMTs) or histone deacetylases (HDACs) activate a variety of intracellular signaling pathways.[24] These have impact on the coordinated expression of multiple genes. There has been interest in the use of HDAC inhibitors to activate the expression of mRNAs that are down regulated in various neuropsychiatric disorders.[24]

There are fundamental contributions on genetics and gene-environment interactions in the field of neuropsychiatry from India. However, there are major lacunae in genomics, proteomics, biomarkers, drug discovery and development of animal models for neuropsychiatry disorders is a major thirst all over the word. The following are the major contributions from India.

### Pedigree analysis of neuropsychiatric disorders from India

Genetics plays an important role in neuropsychiatric research and practice. It is important to understand the exact gene borne influences, mode of inheritance and establishing relationship between gene product and syndromes. Genetic markers like mini satellites, tandem repeat sequences to establish linkages are of great advantage. Rao *et al*.[25] first conducted a pedigree study on the genetics of affective disorder. Rao *et al*.[25] studied the pedigree analysis of a boy (15-year-old) with psychiatric Illness. They found that maternal cousin has juvenile mania, sister has hysterical hyperventilation and neurotic depression, eldest sibling (20 years) was diagnosed to be MDP Circular currently euthymic; mother has major depression and other five individual in the family has psychiatric Illness. The pedigree analysis emphasized need of genetic identification of neuropsychiatric Illness in the families to identify the candidate genes and their linkage.

### Molecular biology of genes in neurological disorders

There are limited studies on the role of genes in neurological disorders. Punia *et al*.[26] studied the six most commonly reported mutations in *LRRK2* gene among Indian PD patients, using PCR-RFLP method. They analyzed G2019S, R1441C, R1441G, and R1441H mutations in 800 PD and 212 controls, I2012T and I2020T mutations in 748 PD patients. LRRK2 gene encodes Leucine-rich repeat kinase 2, associates with the mitochondrial outer membrane. The authors did not find any of the above mutations, except in one female young onset PD patient who has a heterozygous G2019S mutation. They concluded that LRRK2 mutations may be a rare cause of PD among Indians. Dopaminergic pathway has been widely implicated in the pathophysiology of PD. Juyal *et al*.[27] investigated 20 markers in genes including dopamine receptors DRD1, DRD2, DRD3, and DRD4, and dopamine transporter in PD patients and two independent sample sets. The allelic, genotypic, and haplotypic association of these markers with PD was tested in South Indian and North Indian population. They found that 120 bp duplication marker of DRD4 gene showed promising results with PD in both South Indian and North Indian population sets. *Parkin* gene is implicated in the PD, associated with mitochondrial dysfunction. Parkin gene encodes for Parkin protein (subunit of E3 ubiquitin ligase) involved in the proteosomal degradation. Chaudhary *et al*.[28] analyzed the Parkin mutations in familial and sporadic Parkinson’s disease among Indians. They found that mutation frequency of 8.5% in the Parkin gene among Indian PD patients. They found seven novel point mutations and mutations account for 14.3% familial PD, 6.9% young onset and 5.9% late onset sporadic PD. Two patients had homozygous mutations and one has a compound heterozygote among the 20 PD patients with mutations. Alpha-Synuclein protein is implicated in PD and encoded by *SNCA* gene. Nagar *et al*.[29] investigated the prevalence of G88C, G209A and any other mutation(s) in exons 3 and 4 of the alpha-synuclein gene in Indian patients with Parkinson’s disease (PD). They did not find G88C and G209A mutations or any other mutation in exons 3 and 4 of the alpha-synuclein gene. Ganguli *et al*.[30] compared the APOE*E4-AD epidemiological associations in India and the United States in a cross-national epidemiological study. They observed very low prevalence of AD in Ballabgarh, India, but association of APOE*E4 with AD in Indian and US samples is in similar strength. Neurotransmitter dopamine is involved of in the pathophysiology of schizophrenia disorder.[31–33] Srivastava *et al*.[34] performed genotyping in 215 schizophrenia cases and 215 healthy controls from North India for the 31 potential single nucleotide polymorphism/ variable number of tandem repeat markers from nine candidate genes including the dopamine receptors and metabolizing enzymes (synthesis and degradation). They found nominally significant allelic association in case of the catechol-O-methyltransferase rs362204 -/G (*P*=0.028) marker, nominally significant genotypic associations for tyrosine hydroxylase rs6356 A/G (*P*=0.04) and dopamine beta-hydroxylase rs1108580 A/G (*P*=0.025) following the case-control approach. In dopamine beta-hydroxylase, catechol-O-methyltransferase, and dopamine receptor D (2) genes, several significant haplotypic associations were present. Gupta *et al*.[35] analyzed catechol-O-methyltrasferase (COMT) gene in schizophrenia patients. They found no significant association between SNP rs4680 with schizophrenia. They observed highly significant association of seven COMT marker haplotypes with schizophrenia (CLUMP T4 *P*-value = 0.0001).

The study indicated that the interacting effects within the COMT gene polymorphisms may influence the disease status and response to risperidone in schizophrenia patients. Kumar *et al*.[36] analyzed the regulatory and functional polymorphic DNA markers of serotonergic candidate genes in puerperal psychosis and bipolar affective disorder probands. Structural variations/polymorphisms in genes encoding the serotonin transporter in psychiatric disorders have great importance as serotonergic function alters in these disorders. Chowdari *et al*.[37] observed no significant association between cytosolic phospholipase A2 locus (cPLA2) and schizophrenia, which is earlier shown association with schizophrenia. Semwal *et al*.[8] studied the diallelic/multiallelic polymorphisms in some dopaminergic, serotonergic and membrane-phospholipid-related genes in people of North India. They found gene polymorphisms in two genes among the eight tested associated with schizophrenia; one in the promoter region of the serotonin 2A receptor gene and the other in the tryptophan hydroxylase gene. One new allele for the dopamine transporter gene (with eight repeats, 570-bp size) identified in one individual. Mukherjee *et al*. reported a positive linkage and association for psychosis to the chromosomal position 18p11.2.[38]

Verma *et al*.[39] identified a novel nonsense mutation in the SYNGR1 gene. SYNGR1 gene encodes Synaptogyrin 1 associated with presynaptic vesicles in neuronal cells and is located on the chromosomal position 22q13.1. They analyzed the gene in south Indian populations of schizophrenia and bipolar disorder. They found novel mutation Lsy99Glu and common polymorphisms, SNP-ser97ser and Asn ins/ del in SYNGR1 gene. The authors suggested the common pathways may be operating in schizophrenia and bipolar disorder. Verma *et al*.[40] also found a missense mutation in putative cation-channel gene MLC1 in south Indian schizophrenia and bipolar disorder patients.

There are several key issues that need to be resolved before we consider the clinical use of additional HDAC inhibitors to treat neuropsychiatric disorders such as schizophrenia or unipolar depression.[24]. Recent findings from behavioral, molecular, and bioinformatic approaches that are being used to understand the complex epigenetic regulation of gene expression in brain by drugs of abuse and by stress. The advances made in this area promise to open up new avenues for improved treatments of these disorders.[41]

The studies on candidate gene polymorphisms in a popula tion are useful for a variety of gene-disease association. A number of candidate genes from the monoaminergic pathway in the brain, have been associated with schizophrenia. In this study, diallelic/multiallelic polymorphisms in some dopaminergic, serotonergic and membrane-phospholipid-related genes have been evaluated in a control population recruited from North India. Eight genes tested association with schizophrenia for only two gene polymorphisms, one in the promoter region of the serotonin 2A receptor gene and the other in the tryptophan hydroxylase gene. One new allele for the dopamine transporter gene (with eight repeats, 570-bp size), has not been reported in any population.[8] Ravi Kumar *et al*.[42] reported the catabolism of tryptophan and tyrosine in relation to the isoprenoid pathway in neurological and psychiatric disorders.

The concentration of trytophan, quinolinic acid, kynurenic acid, serotonin and 5-hydroxyindoleacetic acid was found to be higher in the plasma of patients with all these disorders; while that of tyrosine, dopamine, epinephrine and norepinephrine was lower. These observations are highly relevant in understanding the neuropsychiatric disorders.

Fragile X syndrome (FRAXA), caused by the expansion of CGG repeats in the 5’ untranslated region of the fragile X mental retardation 1 (FMR1) gene is one of the most common forms of mental retardation. Due to the CCG repeats FMR1 gene becomes transcriptionally inactive by the hypermethylation. Guruju *et al*.[43] performed Molecular diagnosis using polymerase chain reaction and Southern blot analysis and a modified scale of clinical checklist in Fragile X syndrome patients. They found the clinical check list more reliable and matched molecular analysis.

Thelma *et al*.[44] analyzed the fragile X mental retardation 1 haplotypes among non-fragile X males from different Indian caste-based communities. Inter-community comparisons were made which permitted wider comparisons with other ethnic groups. The analysis was made using STR markers, DXS548, FRAXAC1, and FRAXAC2. They observed wide range of haplotypes, with 7-3-4 #x002B; being modal in Brahmins, Kshatriyas, and Vaishyas. Prasad *et al*.[45] analyzed polymorphisms in NOTCH 4 gene in two independent samples from India and USA, consisting of patients with schizophrenia and their parents. (GAAG)(n), (TAA)(n), SNP1, SNP2, and (CTG)(n) DNA markers are evaluated. Transmission distortion, consistent with a modest association was found among India and USA samples. Cossée *et al*.[46] analyzed mutations in the PQBP1 gene in syndromic and non-syndromic X-linked mental retardation (XLMR). They showed that 21 bp in-frame deletions (c.334-354del(21 bp) are implicated in the Indian population.

### Molecular biology of neuronal cell death involving gene-environment interaction

Neuronal cell death is observed in neurological and neuropsychiatric disorders. Several mechanisms and pathways were proposed for the neuronal cell death in these disorders. Gene-environment interactions play an important role in the regulation of neuronal cell death, here we focusing on the genes involved in neurological disorders. DJ-1 gene associated with early onset of neurological disorder Parkinson’s disease (PD) which coupled with dementia. DJ-1 belongs to a family protein which includes transcriptional regulators, proteases and chaperones.

Saeed *et al*.[47] showed that loss of DJ-1 function by the oxidation and degradation by the proteases linked to sporadic PD. DJ-1 is involved in the oxidative pathway of neuronal cell death through mitochondrial dysfunction. Grx1 helps in the recovery of the mitochondrial function and the neuronal cell survival. The Down regulation of glutaredoxin 1 (Grx1, thiol disulfide oxidoreductase) caused depletion of DJ-1 and translocation of Daxx (death protein) leading to cell death. Grx 1 down regulation leads to loss of mitochondrial membrane potential by oxidizing the thiol groups of voltage dependent anion channel. Estrogen regulates the expression of Grx1 in brain. The authors showed that estrogen restored mitochondrial membrane potential by upregulating Grx 1 SH-SY5Y cells after mitochondrial insult.[48] A gender-based difference present on incidence of PD, females being less prone to the disease. Higher expression of Trx (thioredoxin) in females implicated for less incidence of disease in females. Trx prevents the activation of enzymes involved in the redox driven cell death.[49] Karunakaran *et al*.[50] showed cell specific activation of p38 MAP kinase in substantia nigra neurons leads to the neuronal loss in MPTP model of PD. p38 inhibitor prevented the dopaminergic neuronal cell death in the above model indicating the p38 involvement in PD. Excitatory amino acid Beta-N-Oxalyl amino-L-alanine (L-BOAA), inhibits mitochondrial complex I activity through oxidation of critical thiol groups in mice motor cortex.

Glutaredoxin helps in the recovery of complex 1 by regenerating the protein thiols.[51] In the recovery of complex 1, gamma-glutamyl cysteine synthetase (gamma-GCS), the rate limiting enzyme in glutathione synthesis is upregulated.[52] Aluminum (Al) levels are elevated in the serum samples of fragile X syndrome and also provide evidence for the Latha *et al*.[53] showed that aluminum induced B-Z conformational change in (CCG)12 repeats. Z-DNA conformation is confirmed in (CCG)12-repeats using Z-DNA specific polyclonal antibodies, thermal denaturation, ethidium bromide binding patterns. They studied the conformation of (CCG)_12_ after removing the Al using a chelating drug desferoximine.

Interestingly, they found that even after the removal of Al (CCG)12 repeats remain in Z-DNA conformation. Probably the aluminum which is elevated in Fragile X syndrome people may alter FMR1 gene integrity and altered its expression levels. Mustak *et al*.[54] estimated the trace metals (Na, K, S, Ca, Mg, P, Cu, Fe, Zn, Mn and Al) in the serum samples of 3 bipolar types. Trace metals play a significant role in neurological disorders and involved in the environmental gene interactions. They found that Na, K, P, Cu, Al and Mn are increased significantly (P,0.001) in bipolar I (mania). Na, S, Al and Mn are increased significantly (*P*<0.02) in bipolar II hypomania, while in bipolar II depression, Na, K, Cu and Al are increased (*P*<0.001). Na, Mg, P, Cu, and Al are increased significantly (*P*<0.002) in bipolar V. In all 3 bipolar groups S (*P*<0.00001), Fe (*P*<0.002) and Zn (*P*<0.004) are decreased in all 3 bipolar groups.

### Molecular biology of metabolism of neuropsychiatric drugs

The drugs used for the psychiatric disorders work differently among the patients. Genotype of the individuals affects the response of drugs differently. If genotype of the target molecules for the drugs is known it will be great advantage to the clinician to finalize type of the drug and dosage of the drug. Several people worked on the genotypes of patients for different psychiatric disorders in Indian populations. Cytochrome P450 (P450) enzyme plays an important role drug metabolism in the body. P450 enzymes also present brain and involved in the pharmacological modulation of drugs. Cytochrome P4502D constitutively expressed in the pyramidal neurons of CA1, CA2 and CA3 subfields of hippocampus, cerebral cortex, Purkinje and granule cell layers of cerebellum, reticular neurons of midbrain.[55] Tissue-specific alternative splicing of CYPs results in differential drug metabolism in different tissues, such as brain and liver.[56]

The above studies indicate that Cytochrome P4502D may play a role in the metabolism of psychoactive drugs directly at or near the site of action, in neurons, in human brain. Agarwal *et al*.[57] (2008) showed that in brain, drug alprazolam converted more of alpha-OHALP than 4-OAHLP, while in liver it more 4-OAHLP will be formed than alpha-OHALP. Thomas *et al*.[58] analyzed the response and side-effects of Olanzapine in Indian subjects compared to genetic differences in patients. Ten polymorphic markers from seven genes (dopamine D1, D2, D3 and D4 receptors, serotonin 2A receptor and the drug-metabolizing enzymes (CYP1A2 and CYP2D6) are analyzed as potential predictors of response. The authors found that Baseline weight and a 120 bp deletion polymorphism at the dopamine receptor D4 (DRD4) gene were associated with changes in symptom scores. Channabasavanna and Khanna[59] observed drug amineptine, dopamine reuptake inhibitor in major depression patients and suggested dosage of drug. In 20-30% of schizophrenia patients who were on long-term treatment with typical antipsychotic drugs develop Tardive dyskinesia (TD) an iatrogenic disorder. CYP1A2 enzyme was involved in the metabolism of antipsychotic drugs such as clozapine and olanzapine.

Tiwari *et al*.[60] analyzed the common coding polymorphisms in CYP1A2 their role in susceptibility to TD and schizophrenia. CYP1A2*1F (intron A), rs2472304 and rs3743484 (intron D) and rs2470890 (CYP1A2 1545 C > T) in exon 7 polymorphisms identified which are previously reported. Deshpande *et al*.[61] analyzed the Serotonin receptor gene polymorphisms and their association with tardive dyskinesia among schizophrenia patients from North India. Selective serotonin reuptake inhibitors (SSRIs) generally prescribed for the depression patients and they work by blocking the serotonin transporter. Margoob *et al*.[62] studied the serotonin transporter gene (SERT) polymorphism in Indian population. 57 patients of unipolar depressive disorder were genotyped for SERT gene polymorphism and treated with SSRI (escitalopram, 20 mg/ day). The author divided the patients into two groups - one with the II genotype and other being ss or Is genotype. They showed significant difference in response to SSRI between the groups. They concluded that SERT gene polymorphism may have an influence on SSRI treatment in depression.

## CONCLUSION

The major conclusions are:

- Neuropsychiatric burden is going to be in multifold in the next decade
- There is a great need to apply genetic knowledge base to predict and to manage the brain disorders
- We need to discover biomarkers as early predictions of disease and as a endpoints for therapeutic intervention
- Gene-environment interaction is necessary as an etiological feature to understand disease risk factors
- Molecular genotyping for therapeutic intervention is the need of the hour
- Last, but the least, life style and diet as intervention events for diseases need to be exploited
